# Cork Oak Young and Traumatic Periderms Show PCD Typical Chromatin Patterns but Different Chromatin-Modifying Genes Expression

**DOI:** 10.3389/fpls.2018.01194

**Published:** 2018-08-27

**Authors:** Vera Inácio, Madalena T. Martins, José Graça, Leonor Morais-Cecílio

**Affiliations:** ^1^Linking Landscape, Environment, Agriculture and Food (LEAF), Institute of Agronomy, University of Lisbon, Lisbon, Portugal; ^2^Forest Research Center (CEF), Institute of Agronomy, University of Lisbon, Lisbon, Portugal

**Keywords:** CORK, phellogen, lenticels, lenticular phellogen, 5-mC, H3K4Me3, H3K9me2, H3K18Ac

## Abstract

Plants are subjected to adverse conditions being outer protective tissues fundamental to their survival. Tree stems are enveloped by a periderm made of cork cells, resulting from the activity of the meristem phellogen. DNA methylation and histone modifications have important roles in the regulation of plant cell differentiation. However, studies on its involvement in cork differentiation are scarce despite periderm importance. Cork oak periderm development was used as a model to study the formation and differentiation of secondary protective tissues, and their behavior after traumatic wounding (traumatic periderm). Nuclei structural changes, dynamics of DNA methylation, and posttranslational histone modifications were assessed in young and traumatic periderms, after cork harvesting. Lenticular phellogen producing atypical non-suberized cells that disaggregate and form pores was also studied, due to high impact for cork industrial uses. Immunolocalization of active and repressive marks, transcription analysis of the corresponding genes, and correlations between gene expression and cork porosity were investigated. During young periderm development, a reduction in nuclei area along with high levels of DNA methylation occurred throughout epidermis disruption. As cork cells became more differentiated, whole nuclei progressive chromatin condensation with accumulation in the nuclear periphery and increasing DNA methylation was observed. Lenticular cells nuclei were highly fragmented with faint 5-mC labeling. Phellogen nuclei were less methylated than in cork cells, and in lenticular phellogen were even lower. No significant differences were detected in H3K4me3 and H3K18ac signals between cork cells layers, although an increase in H3K4me3 signals was found from the phellogen to cork cells. Distinct gene expression patterns in young and traumatic periderms suggest that cork differentiation might be under specific silencing regulatory pathways. Significant correlations were found between *QsMET1*, *QsMET2*, and *QsSUVH4* gene expression and cork porosity. This work evidences that DNA methylation and histone modifications play a role in cork differentiation and epidermis induced tension-stress. It also provides the first insights into chromatin dynamics during cork and lenticular cells differentiation pointing to a distinct type of remodeling associated with cell death.

## Introduction

Plants are exposed to adverse environmental conditions like desiccation, freezing, heat injury, mechanical traumas, and disease. Enveloping protective tissues are thus fundamental to plants survival. Epidermis, the protecting tissue in primary tissues, is replaced by the periderm during stems and roots secondary growth (Evert, 2006). An exceptionally thick periderm is found in the cork oak (*Quercus* *suber* L.), the commercial cork, which due to several valuable properties, like imperviousness to liquids and insulation, is used for a wide number of important industrial applications (Pereira, 2007). Cork is the result of phellogen (cork cambium) meristematic activity followed by a particular differentiation process, involving cork cells expansion, cell walls suberization and deposition of waxes, ending with cell death and complete emptiness of the cells (Natividade, 1950; Pereira, 2007). In cork oak stems, the phellogen arises in the first year of growth in the subepidermal cell layer (Graça and Pereira, 2004) and continuously produces cork cells throughout the tree’s lifespan accumulating a thick periderm very rapidly. Cork is allowed to be firstly harvested when the stem perimeter reaches the legal size (Oliveira and Costa, 2012). The separation of cork is obtained by the physical rupture of phellogen cells, leading to its death. A new traumatic phellogen is formed after cork extraction by a process of meristematic activation within the exposed non-conducting phloem (Fortes et al., 2004). After nine years of renewed growth, cork is thick enough to be stripped off again from the tree. This process is thereafter cyclically repeated allowing the sustainable exploration of cork-oak trees for more than 200 years. The cork produced by traumatic phellogens (*amadia* cork) has the best characteristics for industrial transformation, as opposed to the first cork divided by the original phellogen. However, even this cork can have widely variable characteristics, presumably due to both environmental and genetic factors, expressed as its industrial “quality." Cork quality is defined by the cork tissue thickness and homogeneity (Silva et al., 2005). The cumulative yearly layers of cork cells are locally crossed at certain points by lenticular channels, named cork pores. These channels are formed by the activity of particular regions of the phellogen, the lenticular phellogen, and are thought to permit gas diffusion between the inward living tissues, and the external environment. Cork porosity, meaning the number, dimension, and distribution of lenticular channels is widely variable in corks from different trees (Graça and Pereira, 2004). Corks with high levels of porosity strongly depreciate its industrial and economic value.

DNA methylation, post translational modifications of histones (HPTMs) and RNA-directed DNA methylation (RdDM) are hallmarks in modifying the functional state of chromatin, and together with nucleosome remodeling can alter the nuclear architecture during plant cell differentiation [reviewed in (Pikaard and Scheid, 2014; Ikeuchi et al., 2015; Takatsuka and Umeda, 2015; Latrasse et al., 2016)]. Plant genomes are methylated in CG, CHG, and CHH contexts which requires the activity of specific DNA methyltransferases (DNMTs) DNA METHYLTRANSFERASE 1 (MET1) maintains CG methylation; CHROMOMETHYLASE 3 (CMT3) maintains non-CG methylation in a self-reinforcing loop between histone H3K9 methylation and DNA methylation by requiring KRYPTONITE (KYP/SUVH4), SUVH5, and SUVH6, a H3K9 methyltransferases; and DOMAINS REARRANGED METHYLTRANSFERASES 1 (DRM1) and 2 (DRM2) responsible for CHH *de novo* methylation through the RdDM pathway [reviewed in (Pikaard and Scheid, 2014)]. CG methylation can result in gene silencing when found in promoter regions or be correlated with moderately high transcription when present within gene body (Zhang et al., 2006; Lister et al., 2008). Non-CG methylation is associated with the transcriptional silencing of transposable elements (TEs) (Cokus et al., 2008). Histone PTMs are also important components of chromatin-level control of gene activity contributing to define distinct chromatin states that modulate the access of transcription machinery to DNA [reviewed in (Kouzarides, 2007)]. Among these modifications, the dimethylation of histone H3 at lysine 9 (H3K9me2) is a highly conserved repressive mark found in heterochromatic regions with a particular role in the silencing of TEs and other repetitive DNA (Bernatavichute et al., 2008). Different HPTMs may be associated with transcriptionally active chromatin such as the trimethylation of histone H3 at lysine 4 (H3K4me3) which is a hallmark of transcription initiation (Roudier et al., 2011; Sequeira-Mendes et al., 2014) specifically accomplished by ATXR3 (Berr et al., 2010; Guo et al., 2010) and ATX3 (Chen et al., 2017). Another modification related to active chromatin is the acetylation of histone H3 at lysine 18 (H3K18ac) mainly found in regions surrounding the transcription start site and associated with transcription enhancers (Wang et al., 2008).

The regulation of specific plant developmental processes involving DNA methylation and HPTMs is well known [reviewed in (Pikaard and Scheid, 2014; Ikeuchi et al., 2015; Takatsuka and Umeda, 2015; Latrasse et al., 2016)]. Few studies were conducted in cork oak such as in pollen nuclei (Ribeiro et al., 2009), revealing an unexpected pattern of marks associated either with gene silencing or activation. Also, DNA methylation levels were correlated with tissue maturity during embryogenesis (Rodríguez-Sanz et al., 2014; Pérez et al., 2015), and with differences in cork cellular characteristics (cork quality traits) which could be directly related to original (Inácio et al., 2017) or traumatic phellogen activity (Ramos et al., 2013; Inácio et al., 2017). Notwithstanding the crucial role played by the cork as a protective tissue and its highly valued product, studies on the role of these modifications in cork formation and differentiation are still scarce.

In this work, we used cork oak periderm as a model to seek for the first insights into the formation and differentiation of secondary protective tissues at the chromatin level. We studied the chromatin organization and nuclei structural changes during cork cells differentiation together with the dynamics of DNA methylation and HPTMs in young and traumatic periderms, formed after cork extraction wounding. The relative expression of the corresponding chromatin-modifying genes was compared through qRT-PCR in both periderms. In addition, relationships between gene expression and the most relevant cork quality traits were investigated.

## Materials and Methods

### Sampling

Young periderms were collected from sprigs harvested in randomly chosen cork oak adult trees located in Tapada da Ajuda field, Lisbon: herbaceous ‘just burst’ sprigs (hereafter referred as herbaceous); around one-year-old sprigs slightly lignified (hereafter referred as one-year old); and three-year-old sprigs heavily lignified (hereafter referred as three-year old). This was performed either by harvesting whole sprigs or by peeling off the periderm tissues.

Traumatic periderms in the form of cork planks were extracted from selected cork-oak trees during the harvesting season. The planks were harvested at breast height (at 1.30 m height from soil) from two adult trees located at Tapada da Ajuda field, Lisbon and seven adult trees located at a cork oak stand (*montado*) in Herdade dos Leitões, Montargil, Portugal. Trees were selected based on their cork quality parameters previously characterized (Inácio et al., 2017). All tissues were harvested during the period of more intense phellogen activity between July and September 2016. Samples of phellogen with contiguous differentiating tissue were collected by scraping the inner surface of cork planks and stored in liquid nitrogen until further use. Small pieces were also cut from the inner surface of the same cork planks and fixed for histologic and cytogenomic analysis. Neighboring cork planks from the same trees were collected for quality traits assessment [described elsewhere (Inácio et al., 2017)].

### Fixation and Sectioning

Immediately after collection, detached periderms were fixed in 4% paraformaldehyde in 1× PBS (phosphate buffered saline: 137 mM NaCl; 0.27 mM KCl; 1 mM phosphate buffer, pH 7.4) under vacuum followed by overnight incubation in fresh fixative at 4°C. Periderms were then dehydrated with a graded ethanol series (50, 70, 85, 95, and 100%), cleared with histoclear (VWR Chemicals), and embedded in paraffin (VWR Chemicals). Tissue sections of 7 μm were made using a microtome and mounted in slides previously coated with poly-L-lysine (1 mg/mL, Sigma-Aldrich, Spain).

To preserve the 3D structure of the nuclei, thick sections of whole sprigs with one and three years old were made using the technique described by Conde et al. (2012).

Traumatic periderm and herbaceous sprigs were fixed in FAA (formaldehyde 37%, acetic acid, ethanol 50%, 1:1:18) under vacuum followed by overnight incubation in fresh fixative at 4°C, and dehydrated with a graded ethanol series (70, 96, and 100%) before embedding in glycol methacrylate – GMA (resin-based product – Technovit^®^ 7100) following the manufacturer’s instructions. Semi-thin sections (2 μm thick) were made using glass knifes on a piramitome LKB Bromma 11800.

### Anatomy and Histology Studies

To identify the structures present in the herbaceous and one-year-old sprigs, these were stained with toluidine blue O (1%), and observed on a Leitz Biomed microscope (Leica Microsystems, Germany) under bright field and photographed with an AxioVision color camera (Carl Zeiss, Germany).

The traumatic periderms were stained with a combination of berberine 0.1% and crystal violet 0.5% to identify the cell walls composition. Preparations were observed in an Axio Imager.Z1 epifluorescence microscope (Carl Zeiss, Germany), and images were acquired with an AxioVision HRm camera (Carl Zeiss, Germany) using the Zeiss filter set 49 (445/50 nm).

The detection of autofluorescence in herbaceous, one-year-old sprigs and traumatic periderms observed with an Axio Imager.Z1 epifluorescence microscope (Carl Zeiss, Germany) using the Zeiss filter sets 49 (445/50 nm), 44 (530/50 nm), and 14 (590 nm) allowed the identification of tissues in the immunodetection experiments.

### Immunolocalization of 5-Methylcytosine and Posttranslational Histone Modifications

Immunodetections of 5-methylcytosine and HPTMs in one and three-year-old sprigs were performed according to (Ribeiro et al., 2009) with some modifications. The antibodies used were chosen since they have been extensively tested in several studies in both plants and animals (Bianco-Miotto et al., 2010; Carvalho et al., 2010; She and Baroux, 2015; Groth et al., 2016) and their specificity has been verified (Nettersheim et al., 2013). Briefly, cell walls were partially digested with 2% cellulose (Sigma-Aldrich, Spain) in 1x PBS and hydrolysable tannins digested with 2 U/ml of tannase (Thermo Fisher Scientific, Waltham, MA, United States). The antigens were further retrieved using the microwave technique at full power for 8 min (Nic-Can et al., 2013). After cooling down, the sections were incubated with 5% BSA (bovine serum albumin, Sigma-Aldrich, Spain) to block non-specific binding, before the incubation with primary antibodies: anti-5mC (1:100 dilution, Abcam AB10805, Cambridge, United Kingdom), anti-H3K9me2 (1:5 dilution, Abcam AB1220, Cambridge, United Kingdom), anti-H3K4me3 (1:50 dilution, Abcam AB8580, Cambridge, United Kingdom), and anti-H3K18ac (1:100 dilution, Abcam AB1191, Cambridge, United Kingdom). Goat polyclonal secondary antibody to mouse or rabbit IgG – H&L conjugated to Alexa Fluor^®^ 488 (Abcam AB150113, AB150077, Cambridge, United Kingdom) were added in a 1:100 dilution accordingly. The slides were mounted in VECTASHIELD Mounting Medium with DAPI (Vector Laboratories, United Kingdom). Thick sections were examined on a Leica SP5 confocal coupled to a Leica DMI6000 (Leica, Germany) using a 63× 1.4 NA Oil immersion objective and HyD detectors in Standard Mode. Laser lines 405 and 488 nm where then used to excite DAPI and Alexa Fluor^®^ 488 fluorochromes with spectral detection adjusted for each. Z-stacks with 0.5 μm were acquired to allow for the identification of entire nuclei.

Immunolocalization of 5-methylcytosine (5-mC) on semi-thin sections of herbaceous sprigs and traumatic periderms was carried out also according to (Ribeiro et al., 2009) but with a prior permeabilization with 1× PBS containing 0.5% Triton X-100. Sections were incubated with mouse monoclonal to 5-mC (Abcam AB10805, Cambridge, United Kingdom), followed by incubation with anti-mouse Cy^TM^3-labeled secondary antibody (Sigma-Aldrich C2181, Spain). The slides were mounted in VECTASHIELD Mounting Medium with DAPI (Vector Laboratories, United Kingdom) and examined with Zeiss Axio Imager.Z1 epifluorescence microscope (Carl Zeiss, Germany) with a 63× 1.25 NA Oil immersion objective. Images were acquired with AxioVision HRm camera (Carl Zeiss, Germany) using Zeiss filter sets 49 (445/50 nm) and 14 (590 nm).

### Fluorescence Intensity Quantification

To achieve precise and reliable comparisons between signals observed at distinct cell differentiation stages, confocal analysis was performed using the same laser excitation and sample emission capture settings (El-Tantawy et al., 2014). The same procedure was applied to epifluorescence analysis on herbaceous sprigs and traumatic periderms preparations. Measurements of the fluorescent signal intensity and nuclei area were performed on the different tissues and/or cork cell layers using Fiji (Schindelin et al., 2019). Projections of maximum fluorescence images from confocal Z-series were obtained and used to quantify fluorescence (Conde et al., 2012). The contour of each nucleus was manually outlined, and the fluorescence intensity was measured (sum of the fluorescence on each pixel within the outlined area). Fluorescence intensity in a non-labeled region was used to normalize all quantifications. The nucleus area was used to normalize the fluorescence intensity to avoid an artificial positive correlation between fluorescence intensity and nuclei size. The ratio fluorescence intensity/nucleus area reflects the amount of DNA methylation or histone modifications. Semi-thin sections of GMA (2 μm) and paraffin (7 μm) led to the segmentation of each nucleus, thus, for statistical analysis, these were grouped in classes according to its area (<15[, [15;25[, [>25 μm^2^). Data are presented in standard boxplots as minimum, first quartile (bottom of box), median, third quartile (top of box), and maximum with the actual spread of individual observations represented by jittered dots. Differences in the ratio fluorescent intensity/nucleus area and in the nuclei area between tissues and/or cork cell layers were tested through Student’s *t*-test and one-way ANOVA followed by Tukey’s multiple comparison test, at a 5% significance level. For non-normally distributed data, Kruskal-Wallis non-parametric and Dunn’s Multiple Correction *post hoc* tests were used. All statistical tests were performed using GraphPad Prism V5.0 software (GraphPad©, San Diego, CA, United States).

### RNA Extraction

Total RNA was extracted from detached periderms and traumatic periderms with the Spectrum^TM^ Plant Total RNA kit (Sigma-Aldrich, Spain) according to manufacturer’s instructions except for some minor modifications: the isolation buffer was supplemented with one volume of Plant RNA Isolation Aid (Thermo Fisher Scientific, Waltham, MA, United States) per unit mass of fresh tissue. Total RNA integrity was assessed by 1% (w/v) agarose gel electrophoresis. mRNA was isolated from total RNA using the Dynabeads mRNA Purification Kit (Thermo Fisher Scientific, Waltham, MA, United States) following the manufacturer’s directions. cDNA was synthesized from 45 ng of mRNA using oligo(dT)_18_ in a 20 μL-reaction volume using RevertAid H Minus Reverse transcriptase (Thermo Scientific, Waltham, MA, United States) according to the manufacturer’s protocol. cDNA was stored at -20°C until further use.

### Putative *Quercus* *suber* Histone Methyltransferases Characterization

The cork oak *Qs*DNMTs (*QsCMT3*, *QsDRM2*, *QsMET1*, and *QsMET2*), and *QsSWC4* evaluated by qRT-PCR in this study were previously characterized (Ramos et al., 2011).

The putative cork oak sequences homologous of characterized histone methyltransferases (HMTs – *QsSUVH4*, *QsATXR3*, and *QsATX3*) were obtained by performing a BLAST at cork oak database ^1^ (Pereira-leal et al., 2014), using the Arabidopsis protein sequences as a query. Complete sequences were retrieved from the cork oak genome version 1.0 (Ramos et al., 2018), except for *QsATXR3*. Protein structure was analyzed using NCBI-CD^2^ (Marchler-Bauer et al., 2016) and SMART searches^3^ (Letunic and Bork, 2017). The potential cork oak *Qs*HMTs orthologous proteins in other angiosperms (**Supplementary Table 1**) were obtained by performing a BLAST at the NCBI database^4^ (Altschul et al., 1997). All sequences were aligned with MUSCLE^5^ (Edgar, 2004) and the alignment was trimmed with GBLOCKS^6^ (Dereeper et al., 2008). *Qs*HMTs were used to perform a phylogenetic analysis with orthologous sequences. Phylogeny analysis was obtained with MEGA 7 software (Kumar et al., 2016), using the maximum likelihood method and a bootstrap of 1000.

### Primer Selection and qRT-PCR Analysis

Seven target genes – *QsCMT3*, *QsDRM2*, *QsMET1*, *QsMET2*, *QsSWC4*, *QsSUVH4*, *QsATXR3*, and *QsATX3* – and four housekeeping genes – *ACT* (actin), *CACs* (clathrin adaptor complexes medium subunit family protein), *GAPDH* (glyceraldehyde 3-phosphate dehydrogenase), *EF-1α* (elongation factor 1-alfa) – were evaluated in this study. Primers were designed using Primer Premier 5.0 software (PREMIER Biosoft International, Palo Alto, CA, United States) except for *QsDRM2* gene and housekeeping genes that was chosen from a previous gene expression studies in cork oak (Marum et al., 2012; Ramos et al., 2013). qRT-PCR experiments were performed in all tissues except for *QsSUVH4* which were only performed in three-year-old sprigs and traumatic periderms. Gene description, NCBI nucleotide and protein sequences accession numbers, primer sequences, and amplicon size are described in **Supplementary Table 2**.

The real-time qPCR was performed in 96 well white reaction plates (Bio-Rad, Hercules, CA, United States), using an IQ5 Real Time PCR (Bio-Rad, Hercules, CA, United States) with at least six individuals and three technical replicates. All cDNA samples were diluted 20-fold and were amplified in triplicate in two independent PCR runs. The reaction mixture was composed of 1 μL diluted cDNA, 0.5 μM of each gene-specific primer and 5 μL master mix (SsoFast EvaGreen Supermix, Bio-Rad, Hercules, CA, United States). The following program was applied: initial polymerase activation, 95°C, 3 min; then 40 cycles at 94°C for 10 s (denaturation), 61°C (except for *ATXR3* which was 55°C) for 20 s (annealing), 72°C 15 s (extension), followed by a melting curve analysis to confirm the correct amplification of target gene fragments and the lack of primer dimmers. No template controls were also included in triplicate for each primer pair. Amplification efficiencies of all genes were estimated with the LinRegPCR quantitative PCR data analysis program (Ruijter et al., 2009) using the raw fluorescence data as input. According to NormFinder algorithm (Andersen et al., 2004) the *GAPDH* and *ACT* genes were chosen as the most stable ones to be used as references. The target genes relative expression ratio was calculated based on amplification efficiencies and expressed in comparison to the geometric mean of reference genes according to (Pfaffl, 2001).

Statistical analysis was performed by clustering data from each group of tissues (young periderms – one and three-years-old – and traumatic periderms) and evaluating statistical differences between them as well as between genes, through Student’s *t*-tests and one-way ANOVA followed by Tukey’s multiple comparison tests, at a 5% significance level.

Also, relationships between DNMTs, HMTs, and *QsSCW4* relative gene expression and cork quality traits previously assessed (Inácio et al., 2017) were analyzed by Pearson’s correlations using ‘rcorr’ function from R environment. The studied traits were porosity coefficient, and pores area, length, and roundness (**Supplementary Table 3**), described in detail elsewhere (Inácio et al., 2017).

## Results

### Cork Cells Keep Their Nuclei Up to the Last Phases of the Differentiating Process

**FIGURE 1:**
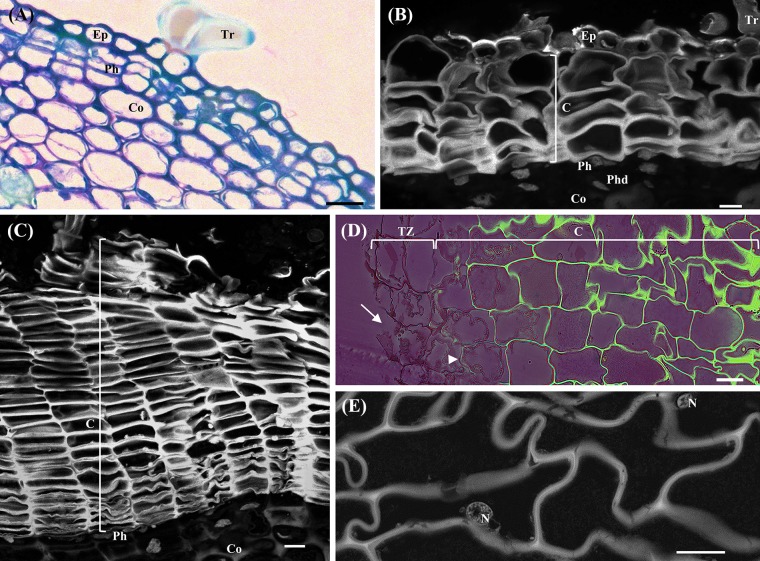
Anatomical analysis of cross-sections of young sprigs (herbaceous, and one and three-year-old sprigs) and traumatic periderms. **(A)** Toluidine Blue O staining of the herbaceous sprigs showing the epidermis with pluricellular trichomes, cortical parenchyma, and the first periclinal divisions originating the precursor of the phellogen. **(B)** Autofluorescence detection under UV light of cork cells and residues of the epidermis with trichomes in cross-sections of one-year-old sprigs. Right below the cork cells is the phellogen, and the underlying tissues phelloderm and cortical parenchyma. **(C)** Autofluorescence detection under UV light of several cork cell layers resulting from the phellogen activity in cross-sections of three-year-old sprigs. **(D)** Berberine/crystal violet staining of traumatic periderms observed under UV light. The cells from the tear zone presented disrupted cellulosic reddish walls (arrows). The contiguous layer of cork cells already shows suberized walls at early stages of differentiation (arrowhead), and highly suberized walls at later stages of differentiation (intense green). **(E)** Cells at later stages of cork differentiation showing entire nuclei. DNA was counterstained with DAPI. Bar = 10 μm. Ep, epidermis; Tr, pluricellular trichomes; Co, cortex; Ph, phellogen; C, cork cells; Phd, Phelloderm; Tz, tear zone; N, nucleus.

In the herbaceous sprigs immediately after burst, only primary growth was detected. From the outside to the inside, epidermis with pluricellular trichomes, cortical parenchyma, primary phloem and xylem, and pith were identified. Periclinal divisions were detected in outermost cortex cell layer establishing the precursor of the phellogen (**Figure 1A**). Different tissues from the outermost to the innermost region of the one-year-old sprigs were clearly recognized: residues of the epidermis with trichomes, few cork cell layers, phellogen, phelloderm, cortex (**Figure 1B**), secondary and primary phloem, secondary and primary xylem, and pith. In the detached periderms the protection tissue is well individualized but the phellogen cell layer was not present since it was torn during the removal. In three-year-old sprigs, due to the increase in thickness of the stem, resulting from the underlying phellogen activity, the epidermis was no longer detectable, and increasing cork cell layers were detected (**Figure 1C**). Cork cell layers exhibited intense autofluorescence when excited with ultraviolet light, indicating the deposition of suberin in their cell walls, which favored the identification of the phellogen, a cell layer without fluorescence right below cork in whole sprigs.

The traumatic periderms, cork planks identical to commercial cork, had the thickness resulting from the nine years growth cycle. In the inner surface of cork planks, the tear zone, i.e., the region where the cork planks were detached from the tree, the cells were disrupted with several contiguous layers of differentiating cork cells already showing suberized walls from the early stages of the differentiation process (**Figure 1D**). Cork living cells showing entire nuclei (DAPI positive) with cytoplasmic content were observed in three-year-old sprigs and traumatic periderms several layers beyond the phellogen and the tear zone (**Figure 1E**). It was impossible to count the total number of living cork cell layers in traumatic periderms, since these were heavily corrugated. The cork cell layers will be referred hereafter as c1, c2, c3, c4, and c5 from phellogen to the outermost layers according to its age, being c1 the cork cell layer most recently formed and c5 the latter differentiation stages.

### Chromatin Condenses, and Nuclei Area Decreases as Differentiation Proceeds in Cork Cells

**FIGURE 2:**
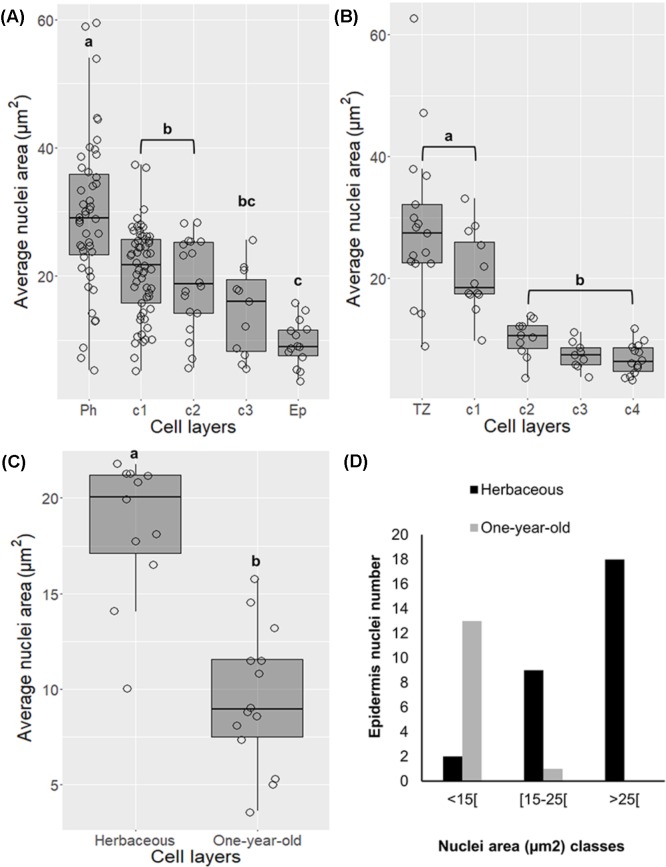
Changes in nuclei area in differentiating cork cells in young periderms from one and three-year-old sprigs **(A)**, in traumatic periderms **(B)**, and in epidermis from herbaceous (entire epidermis) to one-year-old sprigs (disrupted epidermis) **(C)**. **(D)** Epidermis from one-year-old sprigs shows no nuclei with areas higher than 25 μm^2^. Boxplots represent minimum nuclei area, first quartile (bottom of box), median, third quartile (top of box), and maximum. The distribution of every individual measurement is represented by jittered dots.

Alterations in nuclei structure were noticed in differentiating cork cells in both young periderms (one and three-year-old sprigs) and traumatic periderms. Decreases in nuclei area from the phellogen to the cork layers at later stages of differentiation were detected ranging from 2.1-fold in one-year-old sprigs (*p* < 0.001, Tukey’s multiple comparison test, **Figure 2A**) to 4.2-fold in traumatic periderm samples (*p* < 0.001, Tukey’s multiple comparison test, **Figure 2B**). These changes were corroborated by the higher nuclei number with less than 15 μm^2^ found in cork cells at later differentiating stages, particularly in traumatic periderm where no nuclei higher than 15 μm^2^ were found (**Supplementary Figure 1**). The reduction in area was accompanied by drastic changes at the chromatin level. Chromatin progressively condensed as cork cells became more differentiated, and the condensed chromatin preferentially localized at the nuclear periphery.

Nuclei area in epidermis also suffered changes in morphology by decreasing its area from entire to disrupted stage (from herbaceous to one-year-old sprigs) (**Figure 2C**, *p* < 0.001, Tukey’s multiple comparison test), as denoted by the absence of nuclei with areas higher than 25 μm^2^ in the latter (**Figure 2D**).

### Nuclear Fragmentation Is Present in Lenticels of Older Periderms

**FIGURE 3:**
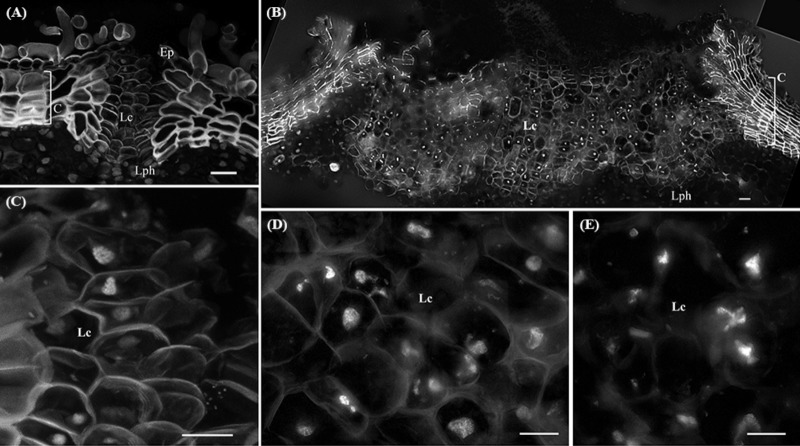
Lenticels found in one-year-old sprigs **(A)** and in three-year-old sprigs **(B)**. The lenticels show a higher number of cell layers above the lenticular phellogen that propel the epidermis upwards causing it to fracture [arrowhead in **(A)**]. In three-year-old sprigs **(B)**, the lenticular phellogen localized in much larger areas due to anticlinal divisions and continuous activity during sprig development. The walls of the lenticular cells emit less autofluorescence than cork cells when excited with UV light, indicative of distinct cell wall composition. **(C)** Nuclei from younger lenticels are whole and round, while from older lenticels are misshapen and fragmented at the innermost regions of the lenticels **(D)**. **(E)** At the outermost regions of the lenticels nuclei are highly fragmented as cells are differentiating with portions of chromatin protruding from a central less condensed chromatin mass. DNA was counterstained with DAPI. Bars in **(A,B)** = 20 μm and in **(C–E)** = 10 μm. Ep, epidermis; Lph, lenticular phellogen; C, cork cells; Lc, lenticular cells.

In younger periderms from one-year-old sprigs, small areas with intense meristematic activity denoted the lenticular phellogen. This meristem produces huge number of cells known as filling tissue forming the lenticels. The high number of cell layers produced by the lenticular phellogen propels the epidermis upwards causing its fracture (**Figure 3A**). In three-year-old sprigs, the lenticels were larger due to continuous lenticular phellogen activity during sprig development (**Figure 3B**). The wall of the cells produced by lenticular phellogen emitted much less autofluorescence than cork cells, indicative of a low suberin content, and a distinct cell wall composition. Contrastingly to cork cells, nuclei from older lenticels (from three-year-old sprigs) became misshapen (**Figure 3C**), and highly fragmented as cells differentiate, with portions of chromatin protruding from a central less condensed chromatin mass to the outermost regions of the lenticels (**Figure 3D**).

### DNA Methylation and Posttranslational Histone Modifications Are Highly Dynamic During Cork Cells Differentiation

To assess the nuclear distribution of well-known markers of different chromatin functional states, immunolocalization of 5-mC and H3K9m2 (repressive marks), and H3K4me3 and H3K19ac (active marks) were performed. These studies revealed differences in the intensity and distribution patterns at distinct tissues and differentiation stages.

**FIGURE 4:**
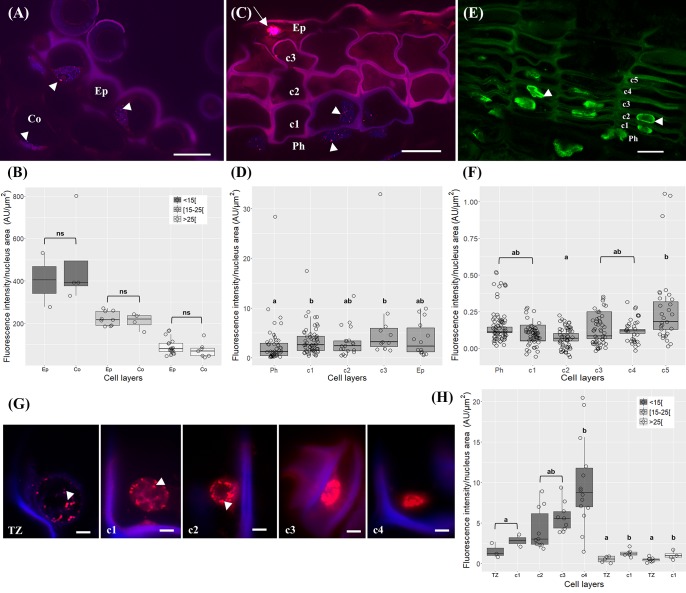
Immunolocalization of 5-methylcytosine (5-mC) in periderms from young sprigs and traumatic periderms. **(A)** Herbaceous sprigs nuclei show discrete 5-mC signals distributed in small spots all over the chromatin in cortex and epidermis, with similar intensity level in all the nuclei area classes [<15[, [15;25[, and [<25[ μm^2^ **(B)**. **(C)** Nuclei of one-year old sprigs show 5-mC signals dispersed throughout the nucleus in all cork cell layers c1, c2, c3, c4; an epidermal nucleus shows high levels of DNA methylation (arrow); **(D)** all cork cells show significant higher intensity levels of 5-mC when compared with phellogen, except for c2 and a 2.5-fold increase is detected from the phellogen to the older cork cell layer (c3). **(E)** Nuclei of three-year-old sprigs show highest intensity of 5-mC signals at the nuclear periphery with a significant increase from c2 to c5 **(F)**. **(G)** Nuclei from traumatic periderms show increase and change in distribution: the nuclei from the tear zone and the most recently formed cork cell layer (c1) display slight dispersed 5-mC signals, that become stronger at the nuclear periphery in older cork cell layers (c2, c3, and c4). **(H)** A significant and pronounced increase in the 5-mC levels is noticed from the tear zone to the older cork living cell layer (c4) in nuclei with areas less than 15 μm^2^. Boxplots represent minimum fluorescence intensity/nucleus area, first quartile (bottom of box), median, third quartile (top of box), and maximum. The distribution of every individual measurement is represented by jittered dots. Arrowheads indicate 5-mC signals. Similar small letters indicate no significant differences. DNA was counterstained with DAPI. Bars in (**A,C,E**) = 10 μm and in **(G)** = 2 μm. Ph, phellogen; Ep, epidermis; Co, cortex; Tz, tear zone.

In very young herbaceous sprigs the nuclei showed discrete 5-mC signals distributed in small spots all over the chromatin both in cortex and epidermis (**Figure 4A**) with similar intensity level (*p* > 0.05, Unpaired *t*-test with Welch’s correction, **Figure 4B**). In one-year-old sprigs, 5-mC signals were dispersed throughout the nucleus in all cork cell layers (**Figure 4C**) and significant higher intensity levels were detected in all cork cells when compared with phellogen, except for c2 (*p* < 0.01 for Ph vs. c1, *p* < 0.05 for Ph vs. c2, Dunn’s multiple comparison test, **Figure 4D**). In fact, a 2.5-fold increase from phellogen to the more differentiated cork cell layer (c3) was noticed. Simultaneously, high levels of DNA methylation were found in epidermal nuclei in cells that remained alive (**Figure 4C**, arrow). In three-year-old sprigs, 5-mC signals were dispersed throughout the nucleus in cork cells, however, the highest intensity was observed in nuclear periphery (**Figure 4E**). There was a significant increment in 5-mC intensity from c2 to c5 at the latter stages of differentiation (*p* < 0.05, Dunn’s multiple comparison test, **Figure 4F**). In traumatic periderm, the tear zone, which locates at the immediate vicinity of the phellogen, showed faint 5-mC fluorescence signals distributed as very small spots all over the chromatin that became more intense at nuclear periphery in the more differentiated cork cells (**Figure 4G**). In nuclei with areas less than 15 μm^2^, a remarkable 6.4-fold increase in the 5-mC level from the tear zone to cork cells at later differentiation stages (c4) was detected (*p* < 0.01, Dunn’s multiple comparison test, **Figure 4H**).

**FIGURE 5:**
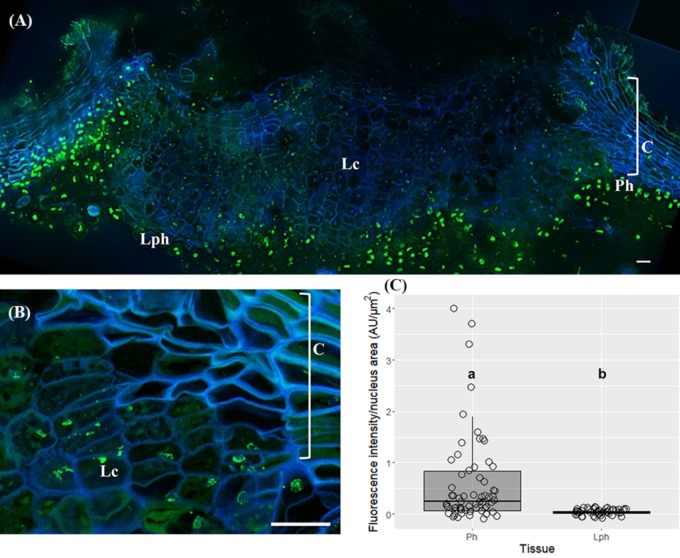
Immunolocalization of 5-methylcytosine (5-mC) in lenticels from three-year-old sprigs. Less intense 5-mC signals in lenticular cells compared with the contiguous tissues like lenticular phellogen, phellogen, cork cells, and cortex **(A)**. **(B)** Fragmented nuclei of lenticular cells showing 5-mC signals. **(C)** Lenticular phellogen exhibit significant lower levels of 5-mC than the contiguous ‘cork’ phellogen; different small letters indicate significant differences. DNA was counterstained with DAPI. Bars = 10 μm. Lph, lenticular phellogen; Ph, phellogen; Lc, lenticular cells; C, cork cells; Co, cortex.

The immunodetection of 5-mC in lenticels exposed differences in fluorescence signal between lenticel filling cells compared to the underlying lenticular phellogen, and neighboring phellogen and cork cells (**Figure 5A**). Although 5-mC signals were distributed throughout the nuclei within lenticels (**Figure 5B**), the quantification of changes in 5-mC levels along lenticular cell layers was unfeasible since nuclei were highly fragmented. Nevertheless, the less intensity in 5-mC signals in lenticular filling tissue compared with the surrounding tissues was quite evident (**Figure 5A**). Furthermore, lenticular phellogen exhibited significantly lower levels of 5-mC than the contiguous cork-forming phellogen (*p* < 0.0001, Unpaired *t*-test with Welch’s correction, **Figure 5C**).

**FIGURE 6:**
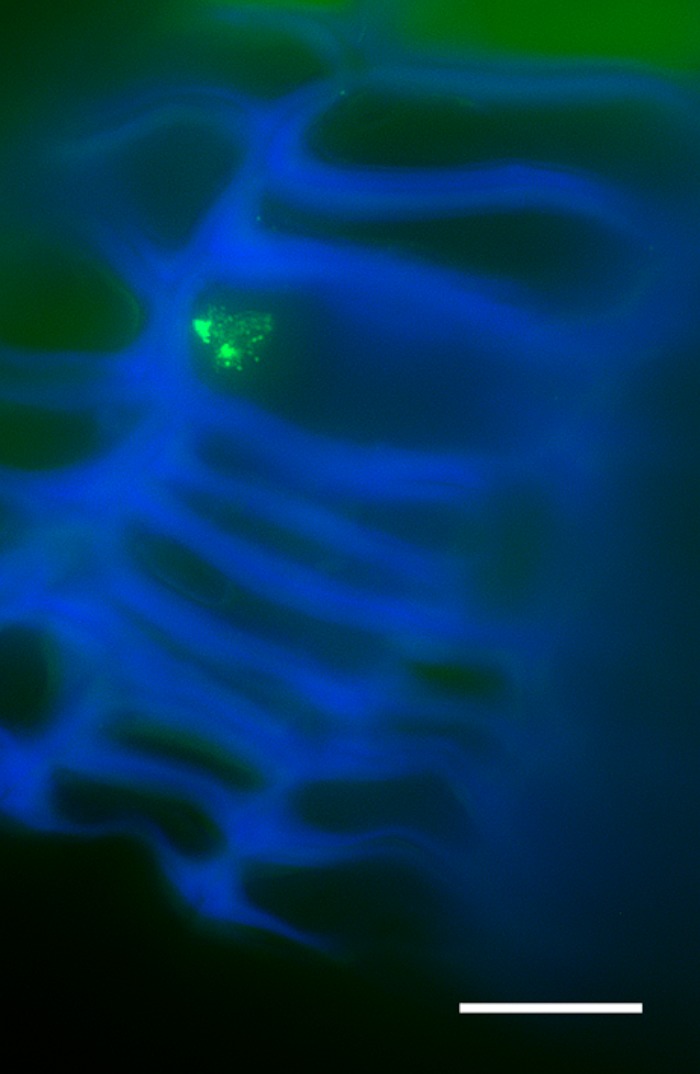
Immunolocalization of histone H3 at lysine 9 (H3K9me2) in cross-sections of one-year-old sprigs shows two condensed chromatin knobs in the periphery of the nucleolus. Bar = 10 μm.

Regarding the dimethylation of histone H3 at lysine 9 (H3K9me2) in one-year-old sprigs, two condensed chromatin knobs in the periphery of the nucleolus could be observed (**Figure 6**). The H3K9me2 signals were not quantified due to the reduced number of labeled nuclei.

**FIGURE 7:**
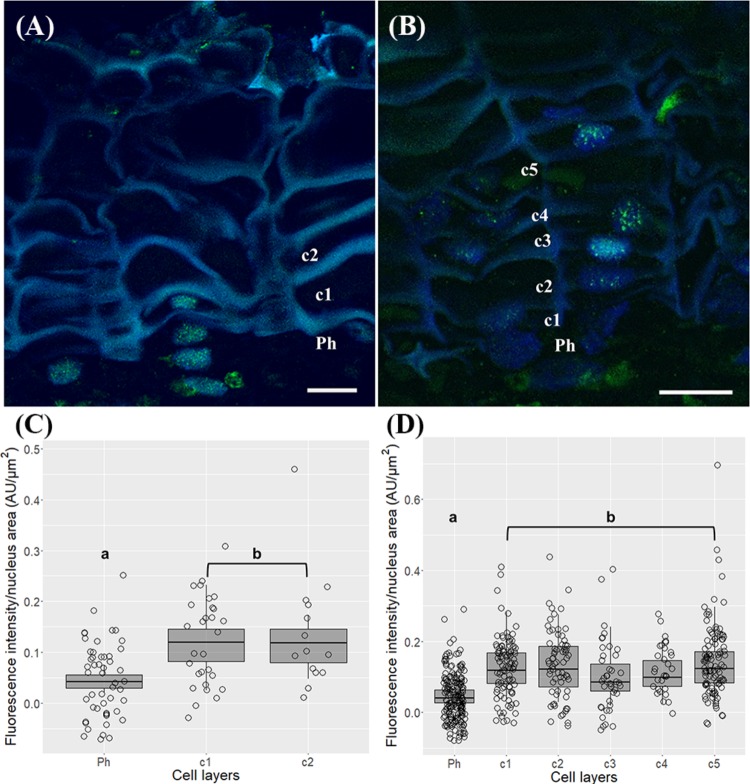
Immunolocalization of the trimethylation of histone H3 at lysine 4 (H3K4me3) in periderms from young sprigs. **(A)** The nuclei from one-year old sprigs show H3K4me3 signals dispersed throughout the nucleus both in phellogen and cork cell layers c1, c2, c3, and c4 with an enrichment from the phellogen to the two contiguous cork cell layers (c1 and c2) with no differences between them **(B)**. Three-year old sprigs nuclei show H3K4me3 signals dispersed throughout the nucleus both in phellogen and cork cell layers c1, c2, c3, and c4 **(C)** with a significant increase from the phellogen to each of the cork cell layers (c1, c2, c3, c4, and c5) and no differences between them **(D)**; similar small letters indicate no significant differences. DNA was counterstained with DAPI. Bar = 10 μm. Ph, phellogen. Boxplots represent minimum fluorescence intensity/nucleus area, first quartile (bottom of box), median, third quartile (top of box), and maximum. The distribution of every individual measurement is represented by jittered dots.

The trimethylation of histone H3 at lysine 4 (H3K4me3) signals were distributed throughout the nuclei both in phellogen and differentiating cork cell layers in one and three-year-old sprigs (**Figures 7A,B**). In one-year-old sprigs, a 2.4 and 2.7-fold enrichment in the level of H3K4me3 was found from the phellogen to the two contiguous cork cell layers, respectively (*p* < 0.001, Dunn’s multiple comparison test, **Figure 7C**), while in three-year-old an average of 2.6-fold increase was noticed from phellogen to each of the differentiating cork cell layers (*p* < 0.001, Dunn’s multiple comparison test), with no differences between them (**Figure 7D**).

**FIGURE 8:**
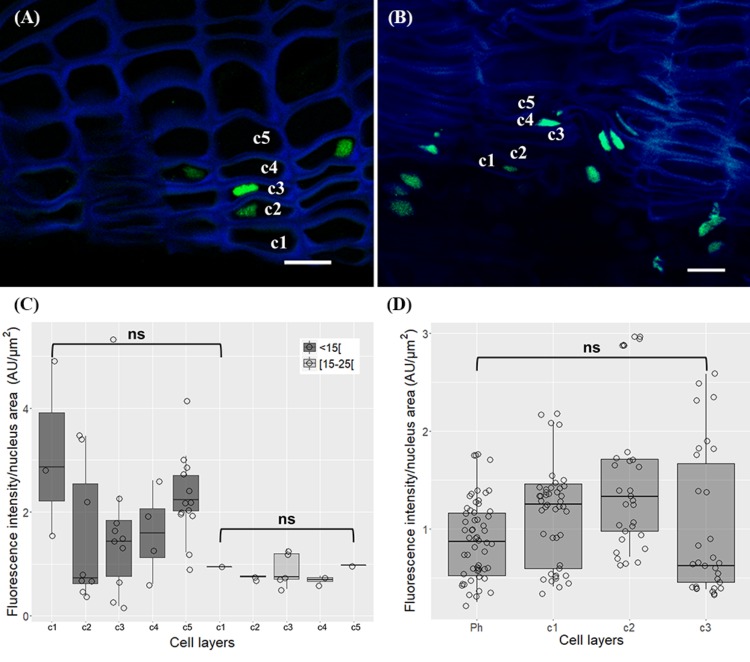
Immunolocalization of the acetylation of histone H3 at lysine 18 (H3K18ac) in in periderms from young sprigs. Nuclei from all cork cell layers (c1, c2, c3, c4, and c5) show dispersed H3K18ac signals in one **(A)** and three-year-old sprigs **(C)**, with no significant differences detected **(B,D)**. DNA was counterstained with DAPI. Bar = 10 μm. Ph, phellogen. Boxplots represent minimum fluorescence intensity/nucleus area, first quartile (bottom of box), median, third quartile (top of box), and maximum. The distribution of every individual measurement is represented by jittered dots.

The nuclear distribution pattern of acetylation of histone H3 at lysine 18 (H3K18ac) revealed nuclei equally and thoroughly labeled in all cork cell layers in one-year-old sprigs (**Figure 8A**) and in all cork cell layers and in phellogen in three-year old sprigs (**Figure 8B**), respectively. No significant differences were detected in the intensity levels (*p* > 0.05 for both sprigs, Tukey’s multiple comparison test, **Figures 8C,D**).

### *Quercus suber* HMTs Proteins Showed All Expected Domains

The *Qs*DNMTs and *QsSWC4* studied in this work were previously characterized (Ramos et al., 2013). The three *Qs*HMTs analyzed revealed that *QsSUVH4* encodes a putative complete protein comprising all four domains found in SUVH4 proteins: SRA-YDG, Pre-SET, SET, and post-SET from N to C terminal; *QsATXR3* encodes a putative partial protein lacking the N-terminal, but with the SET domain of ATXR3 proteins detected at the C terminal; *QsATX3* encodes a putative complete protein, containing all domains described for ATX3 proteins: PWWP, plant homeodomains (PHD) finger, SET, and post-SET from N to C terminal (**Supplementary Figure 2**). All domains seemed conserved in the angiosperms used in this study (see **Supplementary Table 1** for list of angiosperms). Each *Qs*HMT grouped with their orthologous sequences establishing three well individualized groups (**Supplementary Figure 3**), revealing a high degree of conservation.

### HMTs Differential Gene Expression Was Found Between Young and Traumatic Periderms

The relative expression of several *Qs*DNMTs, *Qs*HMTs, and *QsSWC4* was compared in young and traumatic periderms through qRT-PCR.

**FIGURE 9:**
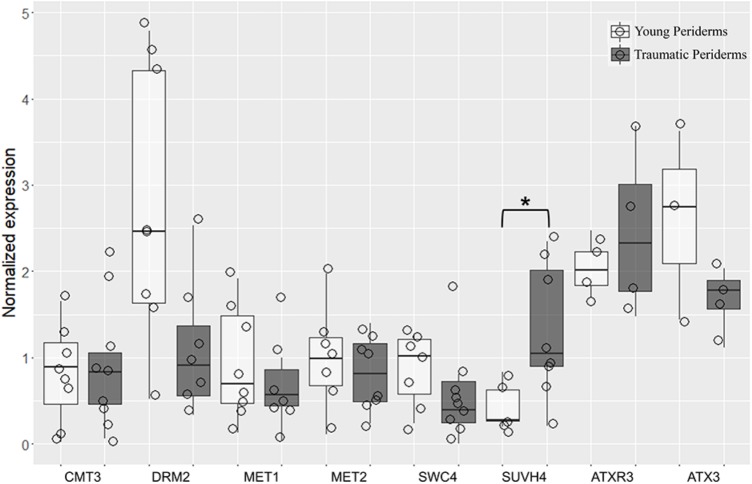
Quantitative real-time PCR (qRT-PCR) evaluation of *QsCMT3*, *QsDRM2*, *QsMET1*, *QsMET2*, *QsSWC4*, *QsSUVH4*, *QsATXR3*, and *QsATX3* mRNA transcripts at different stages of periderm development: young (one and three-year-old sprigs) and traumatic periderms. Results are expressed as means ± standard deviation of at least six individuals and three technical replicates. Transcript levels were normalized to *actin* and *GAPDH*. Asterisks indicate the significance of the difference between young and traumatic periderms. Boxplots represent minimum relative expression, first quartile (bottom of box), median, third quartile (top of box), and maximum. The relative expressive of every individual is represented by jittered dots.

A high accumulation of transcripts was found for *QsDRM2*, *QsMET1*, *QsATXR3*, and *QsATX3* genes in both young and traumatic periderms. Although variability was found between individuals, *Qs*DNMTs, *Qs*HMTs, and *QsSWC4* showed a tendency to be expressed at lower levels in traumatic than in young periderms, except for *QsSUVH4* and *QsATXR3* (**Figure 9**). *QsSUVH4* was amongst the most expressed genes in traumatic periderms, but significantly down-regulated in the young ones (*p* = 0.03, Mann Whitney test). In the latter, *QsSUVH4* was significantly down-regulated when compared with *QsCMT3* (*p* = 0.03, Unpaired *t-*test). The expression of *QsMET1* was positively and significantly correlated with porosity coefficient (*r*≈0.95, *p* = 0.003, ‘rcorr’ R function), while *QsMET2* and *QsSUVH4* expressions showed negative and significant correlations with porosity-related traits (pore length and roundness), one of the most relevant defects found in cork of (*r*≈0.78, *p* = 0.04, ‘rcorr’ R function; **Supplementary Table 4**).

## Discussion

In this work, we used for the first time cork oak periderm as a model to study the formation and differentiation of secondary plant protective tissues and their behavior after traumatic wounding. Periderms are composed of three different types of tissues: the phellogen, a single cell layer secondary meristem with high cell cycling activity; the cork, a multi-cell layer tissue interrupted locally by lenticular channels; and the phelloderm, a single cell layer composed of living parenchymatous cells (Evert, 2006). An integrated approach that combines the immunodetection of several chromatin-modifying marks, and the expression of genes required for the imposition of these modifications enabled a detailed view of the dynamics of active and repressive chromatin marks in nuclei of periderm cells. Also, the establishment of correlations between gene expression and one of the most relevant cork quality traits was achieved. Although periderm formation and development has been extensively studied at the chemical and molecular level [(Miguel et al., 2015; Wunderling et al., 2018) and reviewed in (Franke et al., 2012; Graça et al., 2015; Vishwanath et al., 2015)], our results at the chromatin level brings novelty and adds significant value to the comprehension of its ontogeny.

### Drastic Changes in Chromatin Structure Occurs During Cork Cells Differentiation

The periderm formation in cork oak involves several processes, including cork cells expansion, cell wall suberization (Graça and Pereira, 2004; Pereira, 2007), and likely programmed cell death (PCD) as a terminal differentiation step, resulting in several layers of dead cork cells with empty lumens, with a key function in insulation and protection. During cork cells differentiation, a repressive nuclear compartment was defined by a high level of DNA methylation at the nuclear periphery. Methylation at the nuclear periphery is usually associated with transposable elements (TEs) and repressed genes location (Bi et al., 2017). This suggests a striking reallocation of chromatin within these nuclei in oak meristematic cells since TEs are known to have an interspersed arrangement in potential gene-rich regions, accompanied by a dispersed 5-mC pattern all over the nuclei (Alves et al., 2012). Moreover, different types of nuclei can present different chromatin organizations, as detected by several epigenetic marks patterns in cork oak pollen nuclei (Ribeiro et al., 2009), corroborating a strong chromatin remodeling associated with development and differentiation processes in cork oak. Similar to cork cells differentiation are the changes in nuclear morphology and chromatin organization found amongst the most typical structural features in cells undergoing PCD [reviewed in (Van Hautegem et al., 2014; Latrasse et al., 2016)]. These alterations accompanied by increased levels and a change in the distribution of DNA methylation in differentiating cork cells has also been observed in tapetum cells PCD correlating with an up-regulation of MET1 (Solís et al., 2014), responsible for CpG methylation maintenance in cycling cells (Huang et al., 2010). Indeed, *QsMET1* was amongst the genes with higher levels of relative expression in the cork tissue contradicting, however, previous results in corks with different qualities (Ramos et al., 2013) where was argued that *QsMET2* might be substituting *QsMET1* function to maintain the CpG methylation during phellogen activity. In our work, although at lower levels than *QsMET1* this gene was amongst the genes with the highest expression. DNMT2 or MET2 are known to have weak or no DNA methyltransferase activity although still present in all eukaryotes (Ponger and Li, 2005). For these reasons it has been suggested that, at least in some species, it may have alternative roles (Vieira et al., 2017). In addition, it has been shown that AtDNMT2 interacts with AtHD2s, a unique plant-specific type of histone deacetylase family (Song et al., 2010). Moreover, increase in HD2 expression has been detected during fruit senescence (Kuang et al., 2011), indicating a possible role of DNMT2 in plant cell death programs through the association with histone deacetylases. Therefore, the preferential expression in tissues undergoing PCD, and the other functions might contribute to its high expression. Another important process in meristematic derivative cells is the *de novo* methylation mainly accomplished by DRM1 and DMR2 through the RdDM pathway [reviewed in (Pikaard and Scheid, 2014)]. In differentiating cork cells where DNA methylation is increasing it was not surprising to find the highest levels of gene expression for *QsDRM2*, according to previous results (Ramos et al., 2013). Different methyltransferases act together with chromatin remodeling complexes in an intricate interplay to modify chromatin structure and regulate transcription. CMT3 is required for CHG methylation maintenance, preferring hemimethylated CHG sites (Du et al., 2012), having an active role in TEs silencing (Tompa et al., 2002; Kato et al., 2003; Lippman et al., 2003). Indeed, in traumatic periderms, which are composed of phellogen and contiguous differentiating cork cells, around 20% of methylated CCG *loci* were found (Inácio et al., 2017), although a global DNA methylation view is compromised since the methylation in all other contexts could not be assessed. Considering the emergence of the repressive chromatin domain in these nuclei, and CMT3 function in silencing TEs, its expression in a tissue with intense meristematic activity and in its derivatives, is not surprising.

Epigenetic modifications are read by several protein complexes. DMAP1 is the human homologous of the Arabidopsis SWC4, which is a subunit of the chromatin remodeling complex SWR1C, and also a component of the NuA4 histone acetyltransferase complex, both involved in transcriptional regulation (Bieluszewski et al., 2015) by interacting with the promoters of target genes (Mozgová et al., 2015). SWC4 which has nucleosome acetyltransferase H4 activity (Doyon et al., 2004; Bieluszewski et al., 2015), is involved in several cellular processes such as, the regulation of mitotic cell cycle progression (Shin et al., 2010), DNA repair (Lee et al., 2010), and has been considered essential for plant development (Mozgová et al., 2015). The human DMAP1 is also a co-regulator that stimulates global maintenance of DNA methylation by co-working with MET1 (Rountree et al., 2000) at sites of double strand break repair (Negishi et al., 2009; Shin et al., 2010). The diverse functions of the DMAP1/SWC4 protein, as well as its involvement in two chromatin remodeling complexes associated with gene expression, should account for the expression detected in cork tissues during its differentiation, as already seen in traumatic corks (Ramos et al., 2013).

Like in cork cells, epidermis nuclei decrease in area whereas DNA methylation level increases from herbaceous to one-year-old sprigs, until this cell layer gradually disappear. During the enlargement of stems and roots due to secondary growth, the one-layered epidermis is replaced by the periderm, which thereafter assures the protective role (Evert, 2006). This replacement is caused by internal mechanical pressure driving to epidermis rupture. Thus, epidermis of woody species is a short-lived tissue that undergoes PCD likely as a response to mechanical stress signals, as the changes in chromatin structure observed are typical features of this type of death. Indeed, mechanical stress-induced PCD has also been observed in lateral root cap cells in Arabidopsis driven by the expansion of underlying tissues (Fendrych et al., 2014) or in endosperm breakdown mediated by both endosperm softening and embryo growth (Fourquin et al., 2016). Mechanical stress-activated gene expression has been seen in Arabidopsis (Lee et al., 2005) and poplar (Coutand et al., 2009) in response to mechanic stimuli. Remarkably, columella cells which are subject to tensile stresses when root grows through soil particles, are the most highly methylated cells due to the hypermethylation of TEs (Kawakatsu et al., 2016). Thus, our results point to the modulation of the response to mechanical signals during epidermis replacement through DNA methylation.

In the present work, an enrichment in H3K4me3, a mark associated with gene activation (Roudier et al., 2011; Sequeira-Mendes et al., 2014), was found from the phellogen to all differentiating cork cell layers in young and traumatic periderms, accompanying the PCD process. In a similar process, senescent Arabidopsis leaves showed a strong correlation between up-regulated senescent-associated genes marked with H3K4me3 and gene expression (Hinderhofer and Zentgraf, 2001; Brusslan et al., 2015). Also, in developing secondary xylem of *Eucalyptus grandis*, cell wall-related genes with vital roles in wood formation were found to be H3K4me3-enriched (Hussey et al., 2017). Considering that periderm formation involves cork cells expansion, cell wall suberization and deposition of waxes (Graça and Pereira, 2004; Pereira, 2007), we may speculate that genes involved in suberin and waxes synthesis and deposition, as well as cell death-associated genes are up-regulated in cork cells (Soler et al., 2007; Teixeira et al., 2017; Boher et al., 2018) through H3K4me3 modification. Tri-methylation of histone H3 at lysine 4 is imposed by *ATXR3* and *ATX3* (Berr et al., 2010; Guo et al., 2010; Chen et al., 2017), which level of expression relates well to the amount of H3K4me3 in young and traumatic periderms.

Another important mark associated with transcription is the acetylation of histones. The levels of acetylation of histone H3 at lysine 18 were high, and similar in phellogen and all differentiating cork cell layers. H3K18ac is mainly located in the region surrounding the TSS and associated with enhancers (Wang et al., 2008), pointing to an increase of this mark in highly active cells. H3K18 acetyltransferase has been suggested to be required for the demethylation of a subset of ROS1 (repressor of silencing 1) targets such as the 35S rDNA arrays, and many TEs (Gong et al., 2002; Agius et al., 2006), since these repeats are enriched in this mark (Zhao et al., 2014; Tang et al., 2016). Although the role of H3K18ac in plant cell differentiation is largely unknown we may hypothesize that as cork cells differentiation involve extensive chromatin remodeling, and specific gene expression activation, H3K18ac might be needed to regulate repetitive sequences reorganization through ROS1.

### Cork Differentiation in Young and Traumatic Periderms May Be Under Differential Silencing Pathways

The phellogen in young periderms is formed from cortical cells of primary origin right below epidermis shortly after burst of herbaceous sprigs (Graça and Pereira, 2004), while traumatic periderms are formed through a process of dedifferentiation and meristematic activation of the non-conducting phloem living cells (Evert, 2006). Phellogens with different ages and origins showed distinct DNA methylation profiles apparently linked to aging or/and to traumatic chromatin remodeling ‘memories’ during dedifferentiation (Inácio et al., 2017). High gene expression variability was found between individuals what could be related to their different cork qualities, since DNA methylation polymorphisms were previously found to be associated with distinct phenotypes (Inácio et al., 2017).

Although a close relationship between DNA and histone methylation is evidenced by a self-reinforcing loop between CMT3 and SUVH4 in controlling CHG DNA methylation through H3K9 methylation mark (Cao et al., 2000; Jackson et al., 2004; Huang et al., 2010; Du et al., 2015), *QsCMT3* and *QsSUVH4* expression did not followed the same tendency in young periderms. In fact, *QsSUVH4* expression was detected at significantly lower levels in comparison with *QsCMT3* while in traumatic corks similar levels were detected. *QsSUVH4* putatively contained all canonical domains identified for methylation of H3 at lysine 9 (Rea et al., 2000), including the SET domain and both pre-SET and post-SET domains required for methyltransferase activity (Baumbusch et al., 2001), and the SRA-YDG domain, reader of DNA methylation indispensable for both the interaction with histones and chromatin binding (Citterio et al., 2004). In addition, the phylogenetic analysis with orthologous SUVH4 proteins reinforced the belief of similar functions. All these facts presume the presence of this mark in cork oak nuclei, and although Vičić et al., 2013 were not able to detect H3K9me2 in oak’s cycling cells nuclei, we clearly detected them in cork oak periderms, as previously in vegetative and generative pollen nuclei (Ribeiro et al., 2009). The distinct patterns observed in cork nuclei with two condensed chromatin knobs in the periphery of the nucleolus is in accordance with the enrichment of this mark in condensed and silent rDNA heterochromatic domains (Lawrence et al., 2004), which are preferentially located in nucleoli periphery (Leitch et al., 1992; Silva et al., 2008), and to the role of this mark in the silencing of TEs and other repetitive DNA elements (Bernatavichute et al., 2008). Recent studies on *cmt3* and *suvh4* mutants suggest that these enzymes strongly regulate CHH methylation through a different pathway (Stroud et al., 2013). Also, Zheng et al. (2012) proposed a novel role for SUVH4 in the control of Arabidopsis seed dormancy in a pathway not involving CMT3. The distinct expression patterns of *QsSUVH4* in periderms with different origins, together with different DNA methylation profiles found in traumatic periderms (Inácio et al., 2017), emphasizes the presence of these two silencing pathways in cork oak.

### Cork and Lenticels Show Distinct Chromatin Remodeling Features

The homogeneity of the cork tissue is sometimes interrupted by discontinuities called lenticels that start to develop below stomata in the first periderm through the enhanced activity of the lenticular phellogen (Graça and Pereira, 2004). Lenticels are composed of a loosely filling tissue wherein the cells disaggregate giving rise to the lenticular channels or pores that radially cross the cork, providing a pathway for direct gas exchange between the exterior and the inner tissues (Lendzian, 2006). Although these cells also undergo a PCD program, the process is clearly different from the one suffered by cork cells, since developed lenticels showed highly fragmented nuclei with portions of chromatin protruding from a central less condensed chromatin mass. Very similar chromatin organization and nuclear events were observed during protophloem sieve elements cell death program (Eleftheriou, 1986), and interestingly, in human cells (Dini et al., 1996) pointing to a common pathway in plant and animal kingdoms. Nuclear fragmentation has also been reported in several plant processes such as the ones associated with endosperm degradation during seed development (Wojciechowska and Olszewska, 2003), petal senescence (Yamada et al., 2006), abiotic stress (Ning et al., 2002), post-phloem transport in developing caryopsis (Kladnik et al., 2004), and hybrid lethality (Ueno et al., 2016). Although the features observed in this work are insufficient to comprehend the process underlying lenticular cells disaggregation it is clear that in cork oak periderms there are two types of chromatin remodeling processes associated with cell death: in cork tissue and in lenticels.

The nuclear morphology alterations seen in filling cells nuclei were accompanied by less intense 5-mC signals when compared with the surrounding tissues. This might suggest that other pathways such as the ones involving RNA-mediated gene silencing and/or Polycomb Group (PcG) proteins complexes [reviewed in (Derkacheva and Hennig, 2013; Pikaard and Scheid, 2014; Perera and Goodrich, 2016)] could contribute more than DNA methylation pathways to stably repress genes during lenticel filling cells death. To further test this hypothesis, components of these pathways should be studied in lenticels.

Moreover, the expression of *QsMET1* in traumatic periderms was positively and significantly correlated with porosity coefficient. Since lenticular channels (pores) are composed of cells with low suberin content it is tempting to speculate that this gene might be involved in the silencing of suberin-related genes in these cells. This is in accordance with previous work where this gene was also up-regulated in corks with high porosity (Ramos et al., 2013). Interestingly, differences in DNA methylation were also detected at the phellogen level. Lenticular phellogen showed significant lower levels of 5-mC when compared with cork-forming phellogen, what can be clearly related with the intense meristematic activity of the former when compared with the latter. On the other hand, a negative and significant correlation was found between *QsMET2* expression and pore length, which is supposedly related to regular lenticular phellogen activity throughout the years. *QsMET2* might be acting in cell-cycle genes silencing in lenticular phellogen through its possible interaction with the plant-specific histone deacetylase HD2s (Song et al., 2010). *QsSUVH4* expression and pore roundness showed a negative correlation. A more rounded pore apparently results from an irregular lenticular phellogen activity of all its cells along the years. Therefore, *QsSUVH4* might control genes responsible for its characteristic feature, i.e., higher cell division rates compared with cork-forming phellogen. These results strengthens the previous hypothesis that DNA methylation is likely involved in phellogen cells fate – whether it originates cork cells or lenticel filling cells – and is associated with differences in porosity (Inácio et al., 2017).

## Conclusion

This is, to our knowledge, the first study focusing on the chromatin changes associated with periderm development and wound-periderm formation in trees. Particularly, it offers a comprehensive overview of DNA methylation and HPTMs distribution at the cell level and in distinct cell types. Distinct types of nuclear restructuring processes associated with cell death were also evidenced for the first time during cork and lenticular cells differentiation. Furthermore, this work strengthens the association of DNA methylation with phellogen cells fate and distinct cork quality and suggests that different silencing pathways might contribute to cork differentiation in young and traumatic periderms.

Regardless the limitations of this work in understanding the functional role of these marks in PCD programs, it puts forward a novel view and important breakthroughs into developmental processes in woody species.

To decipher the involvement of chromatin organization and the functional impact in cork differentiation and PCD, multidisciplinary approaches using reverse genetics, deep methylomes, and transcriptomes analyses must be conducted.

Taken together, our findings provide new insights into the dynamics of active and repressive chromatin marks in phellogen activity, cork differentiation and lenticel patterns that determine its industrial quality.

## Author Contributions

VI performed the laboratory work, data analysis, and worked on the original draft of the manuscript. MM contributed to the qRT-PCR experiments. JG conceived the tree sampling, analyzed the data, and critically reviewed the manuscript. LM-C conceived and designed the experiments, analyzed the data, and critically reviewed the manuscript.

## Conflict of Interest Statement

The authors declare that the research was conducted in the absence of any commercial or financial relationships that could be construed as a potential conflict of interest.

## Footnotes

1 http://corkoakdb.org

2 https://www.ncbi.nlm.nih.gov/Structure/cdd/wrpsb.cgi

3 http://smart.embl.de

4 https://blast.ncbi.nlm.nih.gov/Blast.cgi

5 https://www.ebi.ac.uk/Tools/msa/muscle/

6 http://phylogeny.lirmm.fr/phylo_cgi/one_task.cgi?task_type=gblocks
