# Supplementary material for: Cork Oak Young and Traumatic Periderms Show PCD Typical Chromatin Patterns but Different Chromatin-Modifying Genes Expression
Source: Front Plant Sci. 2018 Aug 27;9:1194. doi: 10.3389/fpls.2018.01194 (PMC6120546; doi:10.3389/fpls.2018.01194)

Supplementary Figure 1. Nuclei number in the different cell layers from *amadia* cork sample. Nucleus were grouped in classes according to its area ([<15[, [15;25[, [>25[µm^2) since semi-thin sections of GMA (2 µm) led to the segmentation of each nucleus. TZ – tear zone (the region where the cork planks were detached from the tree); c1, c2, c3, and c4 are the cork cell layers from phellogen to the outermost layers according to its age, being c1 the most recently formed and c5 the older cork cell layer.

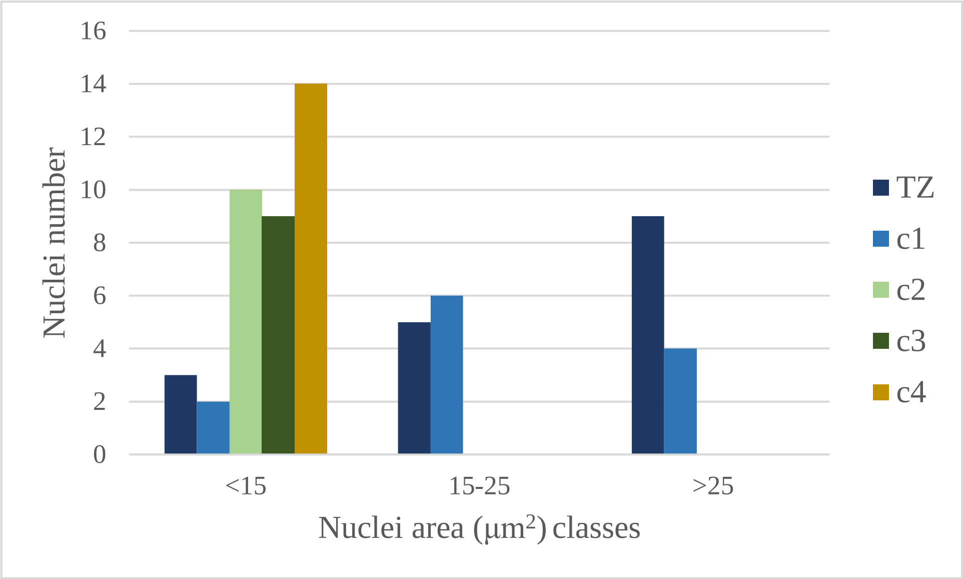

Supplement: Supplementary file 1 [file Image_1.pdf]
