# Supplementary material for: Cork Oak Young and Traumatic Periderms Show PCD Typical Chromatin Patterns but Different Chromatin-Modifying Genes Expression
Source: Front Plant Sci. 2018 Aug 27;9:1194. doi: 10.3389/fpls.2018.01194 (PMC6120546; doi:10.3389/fpls.2018.01194)

Supplementary Figure 2. Diagram of the three *Qs*HMTs structure. **(A)** *QsSUVH4* with four domains found in SUVH4 proteins: SRA-YDG, Pre-SET, SET, and post-SET from N to C terminal; **(B)** *QsATXR3* lacks the N-terminal, but with the SET domain of ATXR3 proteins detected at the C terminal; **(C)** *QsATX3* with the domains described for ATX3 proteins: PWWP, plant homeodomains (PHD) finger, SET, and post-SET from N to C terminal.

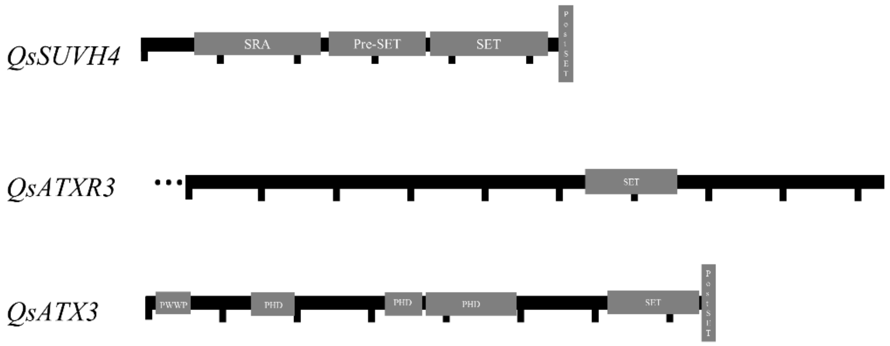

Supplement: Supplementary file 2 [file Image_2.pdf]
