# Supplementary material for: Cork Oak Young and Traumatic Periderms Show PCD Typical Chromatin Patterns but Different Chromatin-Modifying Genes Expression
Source: Front Plant Sci. 2018 Aug 27;9:1194. doi: 10.3389/fpls.2018.01194 (PMC6120546; doi:10.3389/fpls.2018.01194)

Supplementary Figure 3. Phylogenetic tree of *Qs*HMTs and their orthologous sequences. Three well individualized clusters are defined with *Q. suber* proteins grouping with its orthologous sequences (arrows). Tree was constructed on MEGA 7 software, using the maximum likelihood method and a bootstrap of 1000. All protein sequences were aligned with MUSCLE and the alignment was trimmed with GBLOCKS. The protein sequences of angiosperms used in the analysis are listed in Supplementary Table 1.

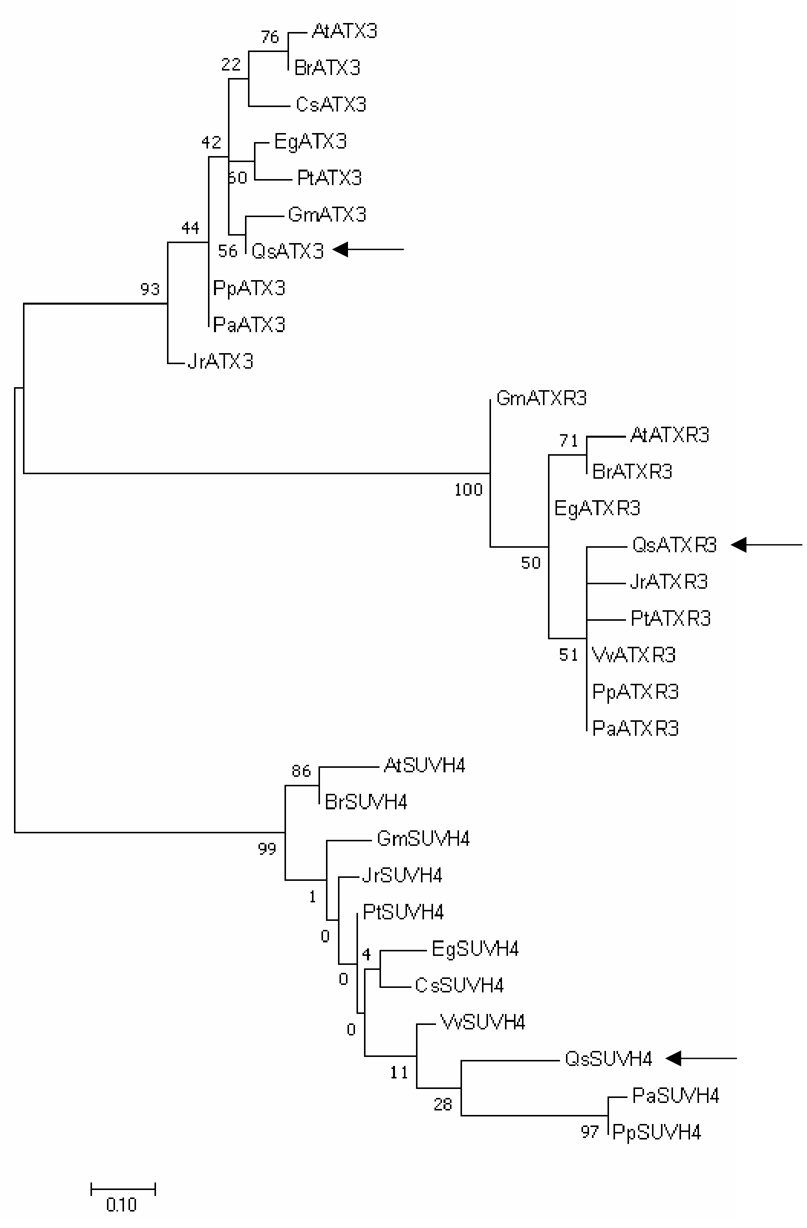

Supplement: Supplementary file 3 [file Image_3.pdf]
