# Supplementary material for: Cork Oak Young and Traumatic Periderms Show PCD Typical Chromatin Patterns but Different Chromatin-Modifying Genes Expression
Source: Front Plant Sci. 2018 Aug 27;9:1194. doi: 10.3389/fpls.2018.01194 (PMC6120546; doi:10.3389/fpls.2018.01194)
Supplement: Supplementary file 4 [file Table_1.pdf]

Supplementary Table 1. Histone methyltransferase sequences used to perform the phylogenetic analysis. For each sequence the name used in this work, the species and the accession number from NCBI (https://www.ncbi.nlm.nih.gov/) are provided.

**Class**	**Sequence name**	**Species**	**Locus**
ATXR3	AtATXR3	*Arabidopsis_thaliana*	NP_193253
	BrATXR3	*Brassica rapa*	XP_009144717
	EgATXR3	*Eucalyptus grandis*	XP_010051270
	GmATXR3	*Glycine_max*	XP_006592400
	JrATXR3	*Juglans regia*	XP_018846411
	PaATXR3	*Prunus avium*	XP_021811206
	PpATXR3	*Prunus persica*	XP_020416287
	PtATXR3	*Populus_trichocarpa*	XP_006372997
	QsATXR3	*Quercus suber*	QS126154.0 (sequence in study)
	VvATXR3	*Vitis vinifera*	XP_010657340
ATX3	AtATX3	*Arabidopsis_thaliana*	NP_001078326
	BrATX3	*Brassica rapa*	XP_009104342
	CsATX3	*Cucumis_sativus*	XP_011656748
	EgATX3	*Eucalyptus grandis*	XP_018728259
	GmATX3	*Glycine_max*	XP_006583237
	JrATX3	*Juglans regia*	XP_018846721
	PaATX3	*Prunus avium*	XP_021815266
	PpATX3	*Prunus persica*	XP_020411934
	PtATX3	*Populus_trichocarpa*	PNT50159.1
	QsATX3	*Quercus suber*	XP_023876913 (sequence in study)
SUVH4	AtSUVH4	*Arabidopsis_thaliana*	NP_196900.1
	BrSUVH4	*Brassica rapa*	XP_009131370
	CsSUVH4	*Cucumis_sativus*	XP_011651591
	EgSUVH4	*Eucalyptus grandis*	XP_010068638
	GmSUVH4	*Glycine_max*	XP_006604199
	JrSUVH4	*Juglans regia*	XP_018834874
	PaSUVH4	*Prunus avium*	XP_021823630
	PpSUVH4	*Prunus persica*	XP_020417219
	PtSUVH4	*Populus_trichocarpa*	XP_021823630
	QsSUVH4	*Quercus suber*	XP_023924619 (sequence in study)
	VvSUVH4	*Vitis vinifera*	XP_010660678
