# Supplementary material for: Cork Oak Young and Traumatic Periderms Show PCD Typical Chromatin Patterns but Different Chromatin-Modifying Genes Expression
Source: Front Plant Sci. 2018 Aug 27;9:1194. doi: 10.3389/fpls.2018.01194 (PMC6120546; doi:10.3389/fpls.2018.01194)
Supplement: Supplementary file 5 [file Table_2.pdf]

Supplementary Table 2 – Description of the target genes with respective nucleotide and protein accession numbers, primer sequences and amplicon size.

Target gene	NCBI nucleotide/ protein accession number	Primer sequence(forward/reverse)	Amplicon length (bp)
QsCMT3 - Chromomethyltransferase 3	XM_024063870/ XP_023919638.1	AATGATTATCCTCTACCTGCTT/ TCATAACTCCCGACCCAAAA	224
QsDRM2 - Domain rearranged methyltransferase 2	XP_023884319.1/ XM_024028551	TCGCTAATGAAGGCGGGTGT/ GTGTAGGGTTGGCAAGGAGG	191
QsMET1 - DNA Methyltransferase 1	XP_023871673.1/ XM_024015905	ACAAGGAGGTCCACCACATA/ AGGTTCGTTGGAAAGGCTAC	164
QsMET2 - DNA Methyltransferase 2	XP_023891955.1/ XM_024036187	TCCCTTTGAAGAATGTTG/ GATGCCTGTTGCTACCTG	224 (Ramos et al., 2013)
QsSWC4 - SWR1-complex protein 4	XP_023927278.1/ XM_024071510.1	CCTAAGCGTTCAAACCGTGGTG/ CTGGGCTGGCGATGATGGAG	141
QsSUVH4 - H3K9 Histone-lysine N-methyltransferase	XP_023924619/ XM_024068851.1	CAACGTCCCTTGGTTCAACT/ TGCGATGTTCTACCTGTTGC	211
QsATXR3 - Histone-lysine N-methyltransferase	QS126154.0 (http://corkoakdb.org)	TCACAATTAGGACGGCATGA/ ATGGGATTCGATCTTTGCAG	172
QsATX3 - Histone-lysine N-methyltransferase	XP_023876913/ XM_024021145.1	GGACAGATATCAGGGGCAGA/ GCCGTCACAGGGTCTTGTAT	235
